# Characteristics, prognosis, risk factors, and management of recently diagnosed ductal carcinoma in situ with microinvasion

**DOI:** 10.1002/cam4.4263

**Published:** 2021-09-21

**Authors:** Chunyan Li, Yilan Yang, Jiangfeng Wang, Kairui Jin, Zhaozhi Yang, Xiaoli Yu, Xiaomao Guo, Xingxing Chen

**Affiliations:** ^1^ Department of Radiation Oncology Fudan University Shanghai Cancer Center Shanghai China; ^2^ Shanghai Medical College Fudan University Shanghai China

**Keywords:** breast‐conserving treatment, characteristics, DCISM, mastectomy, prognosis, radiotherapy

## Abstract

**Background:**

Ductal carcinoma in situ with microinvasion (DCISM) represents ~1% of all breast cancer cases and is arguably a more aggressive subtype of ductal carcinoma in situ (DCIS). Lacking studies with a large population, the survival outcomes of DCISM are still poorly understood and the treatment recommendations remain controversial. This study aims to investigate the long‐term outcome of patients with DCISM, potential risk factors for their prognosis, and the difference of survival between patients treated with breast‐conserving surgery plus radiotherapy (BCT + RT) and mastectomy only.

**Methods:**

In total, 1299 patients from 2008 to 2019 with DCISM were retrospectively retrieved. Clinicopathological features were analyzed. Subgroup analysis was conducted between patients who underwent BCT + RT and mastectomy only. Univariate and multivariate analyses were performed to identify prognostic factors for survival. Differences of survival between two groups were compared using the log‐rank test.

**Results:**

Totally, 1286 patients had follow‐up information, the median follow‐up is 54.57 months, the 5‐year local–regional‐free survival (LRFS), distant metastasis‐free survival (DMFS), and overall survival (OS) were 98.6%, 97.1%, and 99.4%, respectively, two deaths were due to breast cancer. Multivariate analysis identified age <40 (*p *= 0.028) and close margin (≤2 mm) as independent negative prognostic factors for LRFS. No prognostic factors were identified for DMFS and OS. The 5‐year LRFS, DMFS, and OS of patients who had DCIS component ≥5 cm and underwent mastectomy without adjuvant radiotherapy were 100%, 98.4%, and 98.4%, respectively. After propensity score matching (PSM), no survival difference was observed between patients treated with BCT + RT or mastectomy only.

**Conclusions:**

DCISM patients had a good survival, even those with DCIS component ≥5 cm. Patients aged <40 or with close margin (≤2 mm) had a poorer LRFS, but not DMFS or OS. BCT + RT is a feasible choice for DCISM patients.

## Graphical abstract

1. DCISM patients had a good survival, even those with DCIS component ≥5 cm.2. Patients aged <40 or with close margin (≤2 mm) had a poorer LRFS, but not DMFS and OS.3. BCT + RT is a feasible choice for DCISM patients.

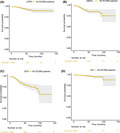

## 1 INTRODUCTION

Ductal carcinoma in situ (DCIS) diagnoses have increased due to increased screening with imaging, and account for about 20% of newly diagnosed breast cancer.^1^, ^2^, ^3^ Among DCIS patients, about 5%–20% are accompanied by micro‐infiltration (namely ductal carcinoma in situ with microinvasion, DCISM). DCISM comprises about 1% of all breast cancer cases.^4^, ^5^ Previous study showed that women are presenting with DCISM more frequently since 2000,^6^ supporting that the management of DCISM is an increasingly common clinical scenario. However, the survival outcomes and optimal treatment recommendations of these patients are unclear.

Previous studies on DCISM from institute‐based data usually had a small sample size (<415) and usually span the pretrastuzumab era. Analysis from the Surveillance, Epidemiology, and End Results (SEER) database was limited by incomplete clinicopathological information and selection bias, making analysis easily affected by many confounding factors unknown, and there were also no records regarding the LRR information. Incidence and survival trends for DCISM have not been reported in a recent population‐based study with a large sample, and difference between breast‐conserving surgery plus radiotherapy (BCT + RT) versus mastectomy has been studied in patients with invasive breast tumors either less than 2.0 cm or 2.1–5.0 cm,^7^, ^8^ but the comparison in DCISM patients has not been reported. However, women with DCISM underwent mastectomy more often than those with DCIS and more frequently than those with higher stage invasive disease (stage 1 breast cancer).^6^, ^9^, ^10^ Therefore, in this context, we investigated the long‐term outcome and risk factors of survival in patients with DCISM that diagnosed between 2008 and 2019. We also present the survival difference between patients treated with BCT + RT versus mastectomy. This study will be important to help physicians better understand the prognosis and biocharacteristics of this relatively rare disease with updated treatment methods, and will be valuable for clinical treatment recommendations for patients with DCISM.

## 2 MATERIALS AND METHODS

### 2.1 Patient and characteristics

The study retrospectively reviewed all the cases of DCISM patients who were treated at our center from 2008 to 2019. A total of 1453 patients were collected, 1299 cases were eligible for further analysis, and 1286 cases had corresponding follow‐up information. The inclusion criteria were: (1) female, aged over 18; (2) primary breast cancer, no distant metastasis at diagnosis; (3) received surgical treatment, including breast‐conserving surgery (BCT) or total mastectomy, with or without sentinel lymph nodes biopsy (SLNB) or axillary lymph node dissection (ALND); (4) pathological diagnosis of ductal carcinoma in situ with micro‐infiltration less than or equal to 1 mm; and (5) adjuvant hormonal therapy, adjuvant chemotherapy, Herceptin treatment, or adjuvant radiotherapy (RT) were offered alone or in combination according to doctor's recommendations, patient's conditions, and willingness. Patients might also receive no further treatments after surgery. The exclusion criteria include: (1) invasive ductal carcinoma with a predominant component of DCIS; (2) patients with a previous diagnosis of invasive breast cancer or other malignant tumors; (3) patients with bilateral breast cancer at diagnosis; and (4) patients who received neoadjuvant chemotherapy. The follow‐up information was collected through outpatient visit records and telephone. The review of data for this investigation was approved by the institutional review board of our center.

### 2.2 Pathology definition

According to the ‘WHO Breast Cancer Histological Classification’ in the fourth edition of 2012, DCISM is defined as DCIS with the extension of cancer cells beyond the basement membrane into the adjacent tissue with no focus more than 1 mm in the greatest dimension. When there were foci more than one, the maximum dimension was used to assess the invasion.^11^ Our study included cases with multi‐foci.

### 2.3 Pathological and immunohistochemical assessment

The status of ER, PR, and Ki‐67 was determined by IHC staining and the status of human epidermal growth factor receptor 2 (HER2) was determined by IHC in combination with fluorescence in situ hybridization (FISH). Cutoff value for ER or PR positivity was at least 1% of tumor cells with positive nuclear staining. The positivity of HER2 refers to the Guideline Recommendations for HER2 Testing in Breast Cancer by American Society of Clinical Oncology/College of American Pathologists in 2007.^12^ HER2/ER/PR positivity in this study is defined as positive for either DICS component or infiltrating component.

### 2.4 Statistical analysis

Local–regional recurrence (LRR) was defined as disease recurrence on the ipsilateral breast or chest wall or the ipsilateral regional lymph node basins (axilla, internal mammary lymph node, supraclavicular fossa, or infraclavicular fossa). Distant metastasis (DM) was defined as breast cancer‐related disease recurrence other than LRR sites. Local–regional‐free survival (LRFS), distant metastasis‐free survival (DMFS), event‐free survival (EFS), overall survival (OS), and breast cancer‐specific survival (BCSS) were calculated from diagnosis. The clinicopathological characteristics between two groups were compared using Pearson's Chi‐squared test or Fisher exact test. Cox proportional hazards models were used to identify prognostic factors of survival. Variables with a *p* value <0.10 in univariate analysis and those previously reported important were included in multivariate analysis. Finally, tumor volume (DCIS component <2 cm vs. ≥2 cm), age at diagnosis (<40 vs. ≥40), axillary lymph node status (pN^+^ vs. pN^−^), margin status (≤2 mm vs. >2 mm), pathological grade (<high grade vs. high grade), index of Ki‐67 (>14% vs. ≤14%), surgery type (BCT vs. mastectomy), chemotherapy status (no vs. yes), and radiotherapy status (no vs. yes) were included in multivariate analysis. Survival differences between two groups were analyzed by Kaplan–Meier method and log‐rank test. Data were analyzed by SPSS 25.0 software, and *p *< 0.05 was thought to have statistical significance.

Subgroup analysis aimed at exploring the survival difference between BCT + RT group and mastectomy without RT (mastectomy only) group. In order to rule out confounding variables between the two groups, propensity score matching (PSM) method was used. PSM was conducted with MatchIt package using R package 3.6.2 (https://cran.r‐project.org/). Variables identified significant in multivariate analysis and those clinically important were included in PSM, including age at diagnosis, tumor size (DCIS component), pathological grade, pathological lymph node status and margin status, the caliper cutoff was set as 0.02, and matching ratio as 1:3.

## 3 RESULTS

### 3.1 Characteristics and overall prognosis

**FIGURE 1:**
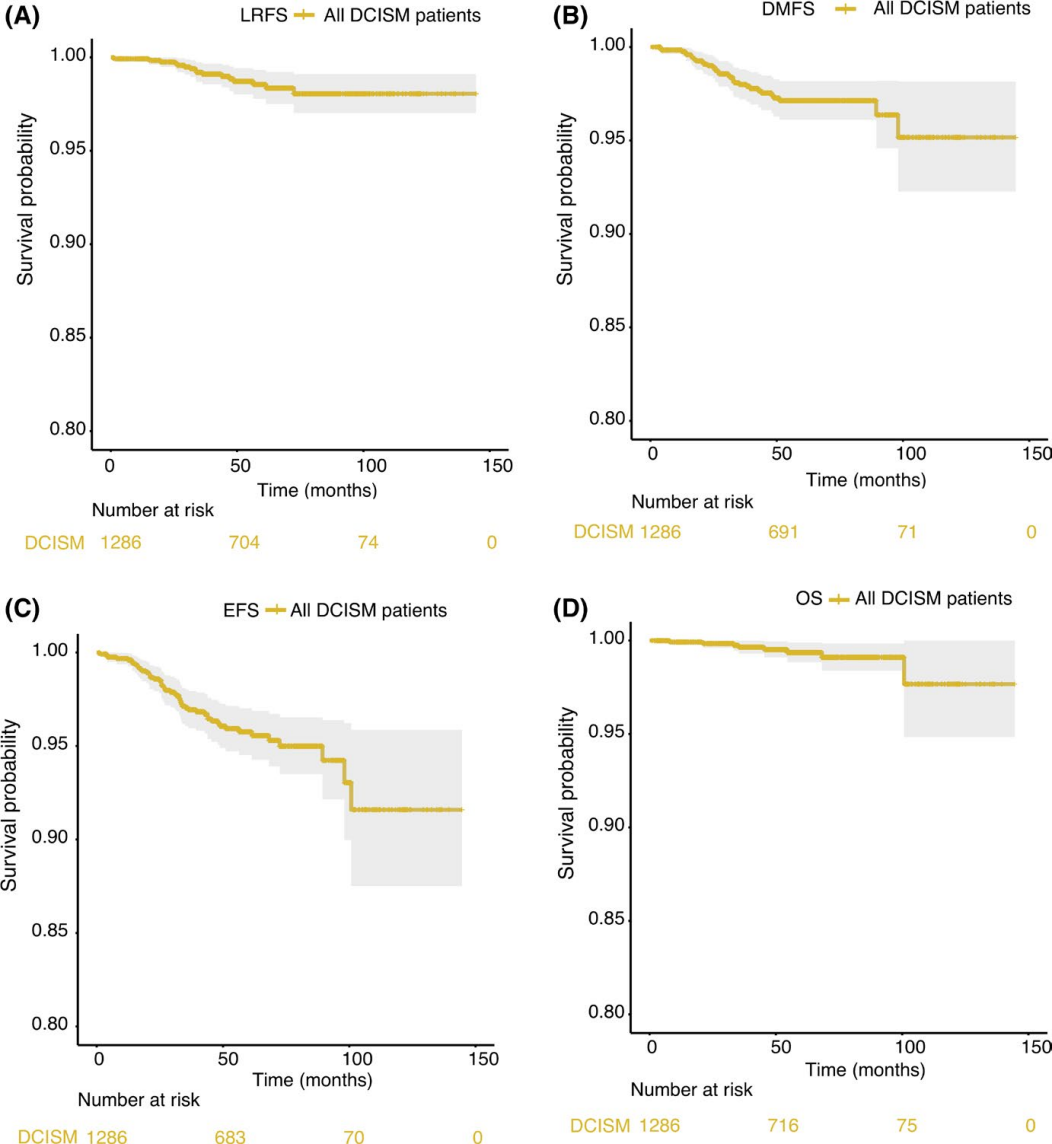
Survival curves for all the DCISM patients in our cohort. (A) LRFS; (B) DMFS; (C) EFS; and (D) OS

Totally, 11.0% (143/1299) of patients received breast‐conserving surgery; 12.4% (161/1299) of patients received adjuvant radiotherapy; 43.9% of patients received adjuvant chemotherapy; 578 out of 628 patients who were ER^+^/PR^+^ received hormonal therapy; and 121 out of 692 patients who were HER2+ received Herceptin treatment. With a median follow‐up of 54.57 (interquartile range 37.3–73.0) months, the 5‐year LRFS, DMFS, EFS, and OS were 98.6%, 97.1%, 95.8%, and 99.4%, respectively (Figure 1). Demographic and clinicopathologic characteristics of the study population are summarized in Table 1.

**TABLE 1:** Patient characteristics

	DCISM (*n* = 1299)	
	No.	%
Age (years)		
Median (range)		
Mean ± SD	49 (22–92) 49.8 ± 10.1	
<40	177	13.6
≥40	1122	86.4
Menstrual status		
Premenopausal	736	56.7
Postmenopausal	559	43.0
Unknown	4	0.30
Surgery		
Breast‐conserving surgery	143	11.0
Mastectomy	1156	89.0
Grade (DCIS)		
<High grade	513	39.5
High grade	686	52.8
Unknown	100	7.70
Pathological tumor size (cm), (DCIS component)		
Median (range) Mean ± SD	2.5 (0.05–13.0) 2.843 ± 1.82	
≤2.0	493	38.0
2.1–5.0	537	41.3
>5.0	105	8.10
Unknown	164	12.6
Pathological lymph node status		
pN^−^	1226	94.4
pN^+^	39	3.00
Unknown	34	2.62
LVI		
Negative	879	67.7
Positive	10	0.80
Unknown	410	31.6
No. of microinvasive foci		
1	834	64.2
≥2	465	35.8
ER		
Negative	644	49.6
Positive	649	50.0
Unknown	6	0.50
PR		
Negative	753	58.0
Positive	540	41.6
Unknown	6	0.50
HER2		
Negative	357	27.5
Positive	769	59.2
Unknown	173	13.3
Ki‐67		
≤14%	272	20.9
>14%	907	69.8
Unknown	120	9.20
Chemotherapy		
No	633	47.6
Yes	584	43.9
Unknown	114	8.6
Hormonal therapy (in HR + patients)		
No	50	8.00
Yes	578	92.0
Anti‐HER2 (in HER + patients)		
No	571	82.5
Yes	121	17.5
Radiotherapy		
No	1136	87.5
Yes	161	12.4
Unknown	2	0.20
Total no. of patients	1299	100.0

Abbreviations: DCISM, ductal carcinoma in situ with microinvasion; ER, estrogen receptor; HER2, human epidermal growth factor receptor 2; LVI, lymphovascular invasion; pN^−^/pN^+^, postoperative lymph node negative/positive; PR, progestogen receptor; SD, standard deviation.

Of the 1265 patients with axillary lymph node assessment, 39 (3.08%) were pN^+^. Among pN^+^ patients, 38 patients had follow‐up information and 2 of them (2/38, 5.26%) had LRR without DM, another two patients had distant metastasis and one of them died from breast cancer.

### 3.2 Patients with LRR

Of the 1286 patients with follow‐up information, 16 patients had LRR and none of them died, 4 of them had distant metastases: one experienced relapse in the ipsilateral axillary lymph nodes 26 months after diagnosis, and developed DM in liver and bones 14 months after LRR; one had LRR and DM simultaneously 31 months after diagnosis, with brain metastasis and multiple lymph node metastases in neck and thorax; another one had contralateral invasive ductal carcinoma 33 months after surgery and suffered ipsilateral supraclavicular lymph node metastases 14 months later; and one had contralateral DCIS 32 months after surgery, with lung and multiple lymph nodes involved in the ipsilateral neck, supraclavicular, axillary, and thorax about 48 months after diagnosis. Among the 12 left patients with LRR events, 8 experienced relapses in the ipsilateral breast, 1 in the axillary lymph nodes, and 3 in chest wall. Fourteen out of sixteen LRR events ( 87.5%) happened within the first 5 years after surgery.

Risk factors of LRFS on univariate and multivariate analyses are shown in Table 2. According to multivariate analysis, age <40 (*p *= 0.028) and close margin (≤2 mm) (*p *= 0.024) were the only two independent negative prognostic factors for LRFS with hazard ratio being 4.127 (95% CI 4.162–14.659) and 10.794 (95% CI 1.361–85.634), respectively.

**TABLE 2:** Risk factors of LRFS on univariate and multivariate analyses (the first variable is the reference)

Variables	HR (95% CI)	*p* value
Univariate BCT versus mastectomy Tumor volume (<2 cm vs. ≥2 cm) Grade (<high grade vs. high grade) Ki‐67 (<14% vs. ≥14%) Age ≥40 versus <40 pN^−^ versus pN^+^ Margin (≤2 mm vs. >2 mm) No‐chemotherapy versus chemotherapy No‐PMRT versus PMRT	0.292 (0.101–0.840) 0.955 (0.321–2.844) 0.813 (0.305–2.169) 1.914 (0.428–8.563) 2.989 (1.038–8.604) 4.845 (1.163–23.260) 4.368 (0.577–33.084) 0.797 (0.297–2.142) 3.003 (1.043–8.645)	0.022 0.935 0.680 0.396 0.042 0.033 0.153 0.653 0.042
Multivariate Age ≥40 versus <40 Margin (≤2 mm vs. >2 mm)	4.127 (1.162–14.659) 10.794 (1.361–85.634)	0.028 0.024

Abbreviations: BCT, breast‐conserving surgery; CI, confidence interval; HR, hazard ratio; LRFS, local–regional recurrence‐free survival; PMRT, postmastectomy radiotherapy; pN^−^/pN^+^, postoperative lymph node negative/positive.

### 3.3 Patients with DM and death

Thirty‐two patients had distant metastasis (32/1286, 2.49%), 20 of them developed contralateral breast cancer afterward, and 12 developed DM in sites other than breast tissue. All DM events happened within the first 5 years after diagnosis. Three patients had simultaneous LRR and have been described before, among the left nine patients with DM events other than breast tissue, three patients developed lung metastases at 4.2, 13.9, and 35.1 months after diagnosis, respectively, and all were alive at the last follow‐up; one patient had distant failure in bone, lung, pleura, liver, and brain 25.57 months after diagnosis and died from breast cancer approximately 2.4 years later; two patients suffered liver metastases, one had brain and bone involvement and developed DM 20.7 months after diagnosis, another one developed DM 17.2 months after diagnosis. Two patients had DM in bone at 12.4 and 26.8 months after diagnosis, respectively. The last one had brain metastasis 16.4 months after diagnosis and died from breast cancer approximately one and a half years later.

Eight patients died and only two (2/8, 25%) deaths were attributed to breast cancer, and both patients developed DM during follow‐up. The 5‐year BCSS was 99.7%. Univariate and multivariate analyses did not identify any risk factors for DMFS and OS.

### 3.4 Survival outcome for patients with DCIS component ≥5 cm

During clinical practice, it is often difficult for doctors to decide on whether to do a chest wall preventive radiotherapy for invasive breast cancer patients who had tumor ≥5 cm, this problem is also happening in DCISM patients with a large mass (DCIS component ≥5 cm), especially those patients with multifocus. Thus, we paid attention on this subgroup of patients.

Totally, 146 patients in our cohort had DCIS component 5 cm or larger, 74 patients had mono‐infiltration while 72 had multifocal infiltration. Among the 72 patients who had multifocal infiltration, 70 patients had LRR information and no one developed LRR, 3 patients had distant metastases and 1 of them died from breast cancer. It is worth noting that the three patients who had DM were all HER2‐positive, the one who developed contralateral DCISM 8 years later received Herceptin treatment while the other two did not. No LRR, DM, or death events were observed among the 74 DCISM patients who had mono‐infiltration.

In patients with DCIS component ≥5 cm, six (6/146) patients were pN^+^ and none had any LRR event while two of them developed DM events afterward: one had axillary lymph node metastases at diagnosis and developed distant metastases in multiple sites and finally died from cancer, another one developed contralateral breast cancer; one (1/146) of the 146 patients had no axillary lymph node assessment at diagnosis, received BCT + RT, and had no event at the last follow‐up; among the left 139 (139/146) patients who were pN^−^, 8 patients underwent mastectomy + RT, and had no subsequent event; 131 patients received mastectomy only, one of them developed lung metastasis 13.9 months after diagnosis and this person was still alive at the last follow‐up.

**FIGURE 2:**
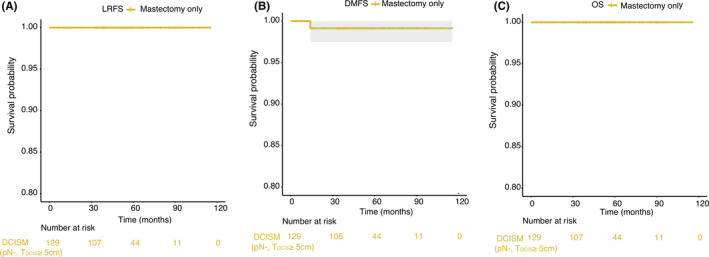
Survival curves for DCISM patients with big tumor (DCIS component ≥5 cm) who were pN^−^ and treated with mastectomy without RT. (A) LRFS; (B) DMFS; and (C) OS

The 5‐year LRFS, DMFS, and OS for patients with large tumors (DCIS component ≥5 cm) who were pN^−^ and treated with mastectomy without PMRT were 100%, 99.1%, and 100%, respectively (Figure 2).

### 3.5 Subgroup analysis (BCT + RT vs. mastectomy without RT)

**FIGURE 3:**
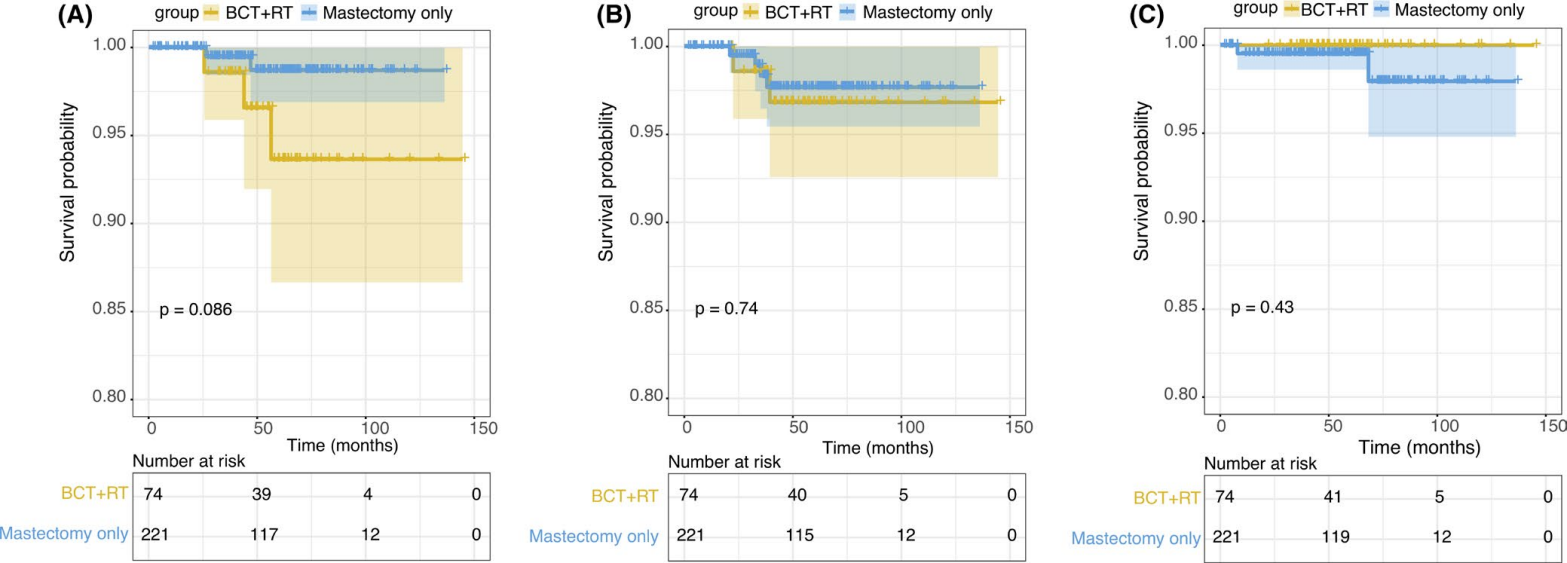
Survival curves for DCISM patients treated with BCT+RT or mastectomy without RT (after PSM). (A) LRFS; (B) DMFS; and (C) OS

A propensity score matching (PSM) was conducted between BCT + RT and mastectomy only groups to remedy confounding factors. After PSM, the baseline characteristics of the two groups are shown in Table 3 and no significant difference was observed on LRFS, DMFS, and OS between the two groups (Figure 3).

**TABLE 3:** Patient characteristics of the two treatment groups (after PSM)

	BCT + RT (*n* = 74)		Mastectomy only (*n* = 221)		*p* value
	No.	% (valid)	No.	% (valid)	
Age Median (range) Mean ± SD	46 (29–68) 46 ± 8.36		49 (24–82) 49 ± 10.86		
<40	19	25.7	56	25.3	0.954
≥40	55	74.3	165	74.7	
Tumor size (cm), (DCIS component) Median (range) Mean ± SD	1.75 (0.2–4.5) 1.785 ± 0.84		1.80 (0.05–13.0) 2.16 ± 1.65		
≤2 cm	52	70.3	155	70.1	0.983
>2 cm	22	29.7	66	29.9	
Grade (DCIS)					
Below high grade	35	47.3	105	47.5	0.975
High grade	39	52.7	116	52.5	
Pathological lymph node status					
pN^−^	73	98.6	218	98.6	
pN^+^	1	1.40	3	1.40	
Margin status					
Close (≤2 mm)	0	0.00	0	0.00	1.000
Negative (>2 mm)	74	100	221	100	

Abbreviations: BCT, breast‐conserving surgery; DCIS, ductal carcinoma in situ; pN^−^/pN^+^, postoperative lymph node negative/positive; PSM, propensity score matching; RT, radiotherapy; SD, standard deviation.

## 4 DISCUSSION

To our knowledge, our study is the largest population‐based study to date investigating the risk factors for LRFS and the survival difference between patients treated with BCT + RT and mastectomy in patients with DCISM, which makes our results valuable for clinical treatment recommendations.

First of all, the basic characteristics in our cohort were mostly similar to other studies reported^13^, ^14^, ^15^ and according to previous reports, the 10‐year LRFS of DCISM patients was about 90.7%, and the DMFS and DFS were about 98.5% and 92.6%, respectively, the RFS was 93%, the 5‐year OS ranged from 91.4% to 99.0%.^5^, ^6^, ^13^, ^16^, ^17^, ^18^, ^19^ In our study, the 5‐year LRFS, DMFS, and OS were similar to or better than previous studies, indicating that DCISM patients generally have a good prognosis. Although most of the DCISM patients lived a long time, a small group of patients suffered from disease recurrence and some even died from breast cancer‐related events. Therefore, it is of great significance to identify risk factors that may be valuable for clinical treatment recommendations.

Lymph node metastasis was often regarded as risk factors for cancer patients. But for DCISM patients, the positivity of lymph node at diagnosis seems to be low. Magnoni et al.^20^ reported an analysis on sentinel lymph node biopsy in 257 women with DCISM patients and found 12.1% of patients had metastatic sentinel lymph nodes. Other studies reported that the pathology positivity rate of lymph nodes of DCISM patients ranged from 2.94% to 7.2%,^14^, ^18^, ^21^, ^22^, ^23^ which was consistent with our study. Among pN^+^ patients with available follow‐up information in our cohort, 5.26% (2/38) had LRR and 5.26% (2/38) had DM; among patients with pN^−^, only 1.07% (13/1214) had LRR and 2.47% (30/1214) had DM.

According to univariate analysis, DCISM patients with pN^+^ seemed to have a worse LRFS (*p *= 0.033), but the *p* value did not reach a statistical significance after multivariate analysis. A study including 322 DCIS/DCISM patients who underwent SLNB concluded that positivity of SLNB in DCIS or DCISM patients was not related to a higher risk of local or distant recurrence.^24^ But the sample size of the study was small, with a median follow‐up of only 47.9 months (range, 0–110.6), and the total number of DCISM patients and number of patients with pN^+^ status were not clearly stated. Limited by the small number of pN^+^ patients in our study, we cannot get a solid conclusion as well, but our data indicated that DCISM patients with pN^+^ disease had relatively higher rate to experience subsequent events than patients with pN^−^ disease (10.53% vs. 3.62% *p *= 0.1049). Recently, a study (*n* = 359) reported that patients with multiple microinvasive foci had worse DFS rate (98.29 vs. 93.01%, *p* = 0.032), however, on multivariate analysis, they found the only independent predictor for worse DFS was axillary metastasis status.^25^ We did not find multiple microinvasive foci to be negatively correlated with either LRFS or DMFS in our cohort, but we do agree that physicians facing DCISM patients with pN^+^ disease should take full consideration with other risk factors together to make the final treatment decision.

Moreover, 5.0% (1/20) of patients in our study with a close margin (≤2 mm) developed LRR, which was only 1.18% (15/1266) in patients with a negative margin (>2 mm). And this factor remained significant on multivariate analysis of LRFS (*p* = 0.024). With the rarity of DCISM patients who had close margin (≤2 mm), we did not find any literatures reporting the influence of margin status on survival of patients with DCISM. However, it has been reported that a close margin after mastectomy may merit radiotherapy to reduce ipsilateral recurrence in DCIS patients,^26^ indicating a negative role for close margin in the survival of DCIS patients. Therefore, we also recommend postoperation radiotherapy to be considered for DCISM patients with close margin.

Another factor associated with higher rate of LRR and worse LRFS (*p *= 0.028, HR = 4.127) in our study was age. This result was consistent with some previous studies on early breast cancer showing that younger breast cancer patients tended to have a higher rate of LRR.^27^, ^28^, ^29^ EORTC 10853 randomly assigned 1010 patients with DCIS to RT group or no‐RT group after breast‐conserving surgery, and found that patients younger than 40 had a higher risk of LRR (HR1.94; *p *= 0.009).^27^ Additionally, a study in 2019 also showed that for patients with DCIS/DCISM after total mastectomy, age <50 was a risk factor for a higher LRR but not for distant metastasis, and patients aged less than 40 were at higher risk for LRR, with a 10‐year LRR rate of 4.2%.^29^

Since doctors will hesitate whether to recommend postmastectomy radiotherapy for DCISM patients with large tumors (DCIS component ≥5 cm) in clinical practice, therefore, we focused on the survival of these patients who underwent mastectomy but did not receive adjuvant radiotherapy. Our results showed that among these patients who were pN^−^, the prognosis was favorable (Figure 2). Besides, multivariate analysis also did not identify tumor volume as a risk factor for any survival of interest in DCISM patients. Therefore, for patients with big tumors (DCIS component ≥5 cm) who underwent mastectomy, postmastectomy radiotherapy may not be necessary. Big tumor volume of indolent DCIS component might be the reason. However, we noticed that all the three patients with DCIS component ≥5 cm who developed distant metastases were HER2‐positive. Among all the patients with LRR events in the entire cohort, 12 out of 14 (75.0%) were HER2‐positive; and among patients with DM, 17 out of 27 (63.0%) were HER2‐positive. This information might indicate that expression of this oncogene may represent a potential biomarker for DCISM at high risk of LRR and DM. Therefore, for DCISM patients with HER2 positivity, Herceptin treatment should be considered.

In addition, previous studies had demonstrated reduced risk from radiotherapy after BCT in patients with DCIS.^28^, ^30^ Radiotherapy after BCT is already one of the standard treatment for DCIS patients.^31^ But conservative surgery is performed less often in DCISM, and there are no standard recommendations for primary treatment of DCISM patients. Limited by sample size, there was also no studies comparing BCT + RT versus mastectomy for DCISM patients, so we did a comparison between the two methods. According to univariate analysis in our study, patients underwent breast‐conserving treatment (BCT) had a relatively poorer LRFS than patients underwent mastectomy. However, we found a higher proportion of patients who had close margin (≤2 mm) (9.8% vs. 0.5%, *p *< 0.001) or aged <40 (21.7% vs. 12.5%, *p *= 0.003) in breast‐conserving surgery group than mastectomy group, and a higher proportion of patients who were pN^+^ (12.3% vs. 1.8%, *p *< 0.001) or with close margin (≤2 mm) (6.8% vs. 0.8%, *p *< 0.001) or age <40 (21.7% vs. 12.4%, *p *= 0.001) in RT group than no‐RT group. This phenomenon may explain the results of the univariate analysis. When considering other factors in multivariate analysis, the significant difference of LRFS stratified by surgical method or RT no longer exist. After PSM, no significant difference on LRFS, DMFS, and OS was observed between BCT + RT and mastectomy only group, indicating that for patients with DCISM, BCT + RT is also a feasible choice.

Altogether, like DCIS, patients with DCISM also had a favorable prognosis, even those with tumors (DCIS component) 5 cm or larger. BCT + RT could be a choice for selected patients. For patients with high risk factors (age <40, HER2+, or with close margin [≤2 mm]), enhanced treatment should be considered.

## 5 DATA AVAILABILITY STATEMENT

The data that support the findings of this study are available from the patient records of the Fudan University Shanghai Cancer Center, Shanghai, China; but restrictions apply to the availability of these data, which were used under license for this study, and so are not publicly available. Data are, however, available from the authors upon reasonable request and with permission of the Fudan University Shanghai Cancer Center, Shanghai, China.

## CONFLICT OF INTEREST

The authors declare that they have no conflict of interest.

## ETHICS APPROVAL

The review of data for this investigation was approved by the institutional review board of Fudan University Shanghai Cancer Center.
